# Transcriptomic Differences between Free-Living and Parasitic *Chilodonella uncinata* (Alveolata, Ciliophora)

**DOI:** 10.3390/microorganisms10081646

**Published:** 2022-08-15

**Authors:** Xialian Bu, Weishan Zhao, Ming Li, Wenxiang Li, Shangong Wu, Hong Zou, Guitang Wang

**Affiliations:** 1 State Key Laboratory of Freshwater Ecology and Biotechnology, and Key Laboratory of Aquaculture Disease Control, Ministry of Agriculture, Institute of Hydrobiology, Chinese Academy of Sciences, Wuhan 430072, China; 2 University of Chinese Academy of Sciences, Beijing 100049, China; 3 Protist 10,000 Genomics Project (P10K) Consortium, Institute of Hydrobiology, Chinese Academy of Sciences, Wuhan 430072, China

**Keywords:** *Chilodonella uncinata*, facultative parasitism, transcriptome, metabolism, adaptation

## Abstract

*Chilodonella uncinata* is a facultatively parasitic ciliate, which can opportunistically parasitize on fish gills and fins, and sometimes even cause host mortality. Previous molecular studies of *C. uncinata* mainly focused on genetic diversity and molecular evolution. There are currently no transcriptome reports studying differences between free-living and parasitic *C. uncinata*. We addressed this by sequencing transcriptomes of these two *C. uncinata* lifestyle types using Smart-seq2 and Illumina HiSeq technologies. In total, 1040 differentially expressed genes (DEGs) were identified. Compared with the free-living type, 494 genes of the parasitic type were downregulated and 546 genes were upregulated. These DEGs were identified through BLAST with NCBI-nr, Swiss-Port, and Pfam databases and then annotated by GO enrichment and KEGG pathway analysis. The results showed that parasitism-related genes such as heat shock proteins (HSPs), actin I, and leishmanolysin were significantly upregulated in parasitic *C. uncinata*. The ciliary-related dynein heavy chain also had a higher expression in parasitic *C. uncinata*. Furthermore, there were significant differences in the amino acid metabolism, fatty acid metabolism, lipid metabolism, and TCA cycle. This study increases the volume of molecular data available for *C. uncinata* and contributes to our understanding of the mechanisms underlying the transition from a free-living to a parasitic lifestyle.

## 1. Introduction

Ciliates are considered the most harmful parasites of fish, among which, *Chilodonella* species such as *C. hexasticha* and *C. piscicola* can cause mass mortalities and, thus, substantial economic losses to the freshwater aquaculture and ornamental fish industries [1,2,3,4]. Unlike the two pathogenic obligate species *C. hexasticha* and *C. piscicola*, *C. uncinata* is commonly considered as a free-living ciliate species and has been used as a common experimental organism in many genetic studies, such as chromosome copy number variation [5,6], genome structure evolution [7], protein evolution [8], and protein diversity [9].

However, recently *C. uncinata* was confirmed to be infective to goldfish and can cause the same tissue damage as other *Chilodonella* species [10,11]. A successful infection model of goldfish was established by adding the *C. uncinata* isolates from cultures to the goldfish’s gills [10,11], which has the potential to be used in subsequent diagnostic test and drug screening. This could also be a good model to study the effects of parasitic lifestyles on physiology and metabolism.

With the rapid development of sequencing techniques in recent years, the multiomics combination has also been used in the research of *C. uncinata* [12]. Due to the small size of *C. uncinata* and difficulties in collecting samples, the traditional methods for sequencing are fairly laborious, time consuming, and costly. The emergence of single-cell omics techniques such as single-cell genomics and transcriptomics helped overcome these difficulties. So far, single-cell transcriptomics studies of *C. uncinata* based on the Smart-seq amplification mostly focused on genome architecture [13,14] and genome diversity [13]. However, the improved method, Smart-seq2, which is more sensitive and more accurate [15], has not been applied yet.

In this study, we used the single-cell transcriptome method based on the Smart-seq2 amplification to explore the transcriptomic differences in *C. uncinata* in these two different habitats (free-living and parasitic). In more detail, differentially expressed genes were annotated using NCBI’s nonredundant protein (NR), Swiss-Port and Pfam databases, and GO enrichment and KEGG pathway annotation analyses were performed. Mitochondrial proteins related to the energy metabolism of the two *C. uncinata* were also identified. Studying the transcriptional differences in response to habitat changes will help us to better understand the mechanisms underlying the transition from a free-living to a parasitic lifestyle.

## 2. Methods and Materials

### 2.1. Host Examination and Sample Collection

Our laboratory has established the goldfish (*Carassius auratus*) infection model of *C. uncinata* in the early stage [11]. Goldfish were kept in 100 L opaque aquaria and were fed once a day at 1% body weight with commercial fish pellet feed. The aquarium temperature was adjusted with an air conditioner at 25 ± 1 °C. So in this study, the host used for the parasitic *C. uncinata* collection were the infected goldfish. The fishes were anaesthetized with 0.02% tricaine methanesulfonate (MS-222, Sigma, St. Louis, MI, USA) according to the manufacturer’s instructions. Gills and fins were isolated and examined. The parasitic *C. uncinata* were collected with Pasteur micropipettes and washed three times in distilled water to remove the potential contaminants, then collected in tubes with a lysis component and ribonuclease inhibitor (Anorada, Beijing, China). The free-living *C. uncinata* were collected from the culture medium in the laboratory as described in Bu et al. [11]. To be more specific, 10 g of fresh wheat grains were added to 250 mL ddH_2_O and then sterilized to be prepared as the culture medium. Single ciliate cells in good condition (high motility and large in size) were placed into the medium to be cultured in the dark at 25 °C. The two types of specimens were immediately frozen by liquid nitrogen flash freezer after collection, then stored at −80 °C before next procedure.

### 2.2. Single-Cell Transcriptome Amplification and Sequencing

The single-cell samples were amplified by the Smart-Seq2 method. First-strand cDNA synthesis was performed using an Oligo-dT primer, followed by PCR amplification to enrich the cDNA and magbeads purification to clean up the production. Then, the cDNA production was checked by Qubit^®^ 3.0 Fluorometer (Invitrogen, Waltham, MA, USA) and Agilent 2100 Bioanalyzer (Agilent, Santa Clara, CA, USA) to ensure the expected production with a length of 1~2 kbp. After that, the cDNA was sheared randomly by ultrasonic waves for the Illumina library preparation protocol including DNA fragmentation, end-repair, 3′ ends A-tailing, adapter ligation, PCR amplification, and library validation. After the library preparation, PerkinElmer LabChip^®^ GX Touch (PerkinElmer, Boston, MA, USA) and StepOnePlus™ Real-Time PCR System (Applied BioSystems, Foster City, CA, USA) were performed for library quality inspection. Qualified libraries were then loaded on the Illumina Hiseq platform for PE150 sequencing (Illumina, San Diego, CA, USA).

### 2.3. Transcriptome Assembly and Annotation

Trimmomatic v0.39 [16] was used to filter the Illumina adapters and low-quality sequences. Clean reads were processed with FastQC v0.11.9 [17]. Then, paired-end sequences were assembled using Trinity v2.13.2 [18], and BLAST [19] was used to filter out the sequences that were significantly similar to the bacteria, and then aligned using Bowtie2 v2.2.3 [20] with default parameters. BUSCO v5.2.2 [21] was used to assess transcriptome completeness with the alveolate_odb 10 data set. GC content and N50 were also calculated, and then open-reading frames (ORFs) were predicted and translated from the assembled transcripts using Transdecoder v5.5.0 (http://transdecoder.github.io) (accessed on 1 May 2021). To further maximize sensitivity for capturing ORFs that may have functional significance, BLASTP searches [19] against the Swiss-Port and Pfam databases were conducted. The unigenes were also annotated based on the NR database. Diamond v2.0.12 [22] was used for the BLAST search.

### 2.4. Differentially Expressed Genes (DEGs) Analysis

RNA-seq data were mapped back onto the assembled transcriptome and a gene expression matrix was generated for the differential expression analysis. Differential expression analysis of parasitic *C. uncinata* and free-living *C. uncinata* was carried out by edgeR v3.36.0 [23]. The read count for each predicted gene was normalized as counts per million (CPM). The adjusted *p*-value, i.e., q-value, was used for subsequent analysis. Q-value < 0.05 and |log2 fold change (FC)| ≥ 1 were set as the thresholds for differential expression analysis. The differential expression results were then annotated by GO terms and KEGG pathway enrichment analyses, with the help of the online tool EggNOG-mapper (http://eggnog-mapper.embl.de) (accessed on 1 June 2021) [24] and TBtools v1.09 [25]. All results were visualized in R v4.1.1 [26].

### 2.5. Mitochondrial Proteins Identification

Putative mitochondrial proteins were detected from translated transcripts by BLASTP searches, using the well-described mitochondrial proteomes of *Homo sapiens* [27], *Tetrahymena thermophila* [28], and homologs from other ciliates as queries. All identified mitochondrial proteins were then annotated by the BlastKOALA server (www.kegg.jp/blastkoala/) (accessed on 1 June 2021) [29]. Those with hits were determined as containing mitochondrial-targeting signals based on the predictions from TargetP [30] and MitoFates [31]. To confirm that genes of these identified mitochondrial proteins were of ciliate origin, codon usage analysis and Codon Adaptation Index (CAI) calculation were made by the EMBOSS package [32]. The value of CAI ranges from 0 to 1, where the value of 1 implies that only the most frequent codons were used within a gene, which indicates the strongest codon bias, and 0 means that only the least frequent codons were used [33].

## 3. Results

### 3.1. Transcriptome Sequencing and Assembly Results

Two transcriptomes were sequenced in the present study: a parasitic *C. uncinata* (PCu) and a free-living *C. uncinata* (FCu) (Table 1). After removing adapters, low-quality regions, and potential contaminants, 16,359,946 and 15,592,937 clean reads were obtained, respectively. Reads have been deposited in GenBank under the BioSample number PRJNA842862. Reads have also been deposited in the Genome Sequence Archive (GSA) under the accession number CRA007627. The total length of the contigs was 31,952,883 bp and the number of contigs was 81,820. The maximum contig length was 14,589 bp, with the GC content of 46.74% and the N50 of 742 bp. The completeness of the assembly results was 62.6%. All the assembly results are shown in Table 1.

### 3.2. Differentially Expressed Genes

**Figure 1:**
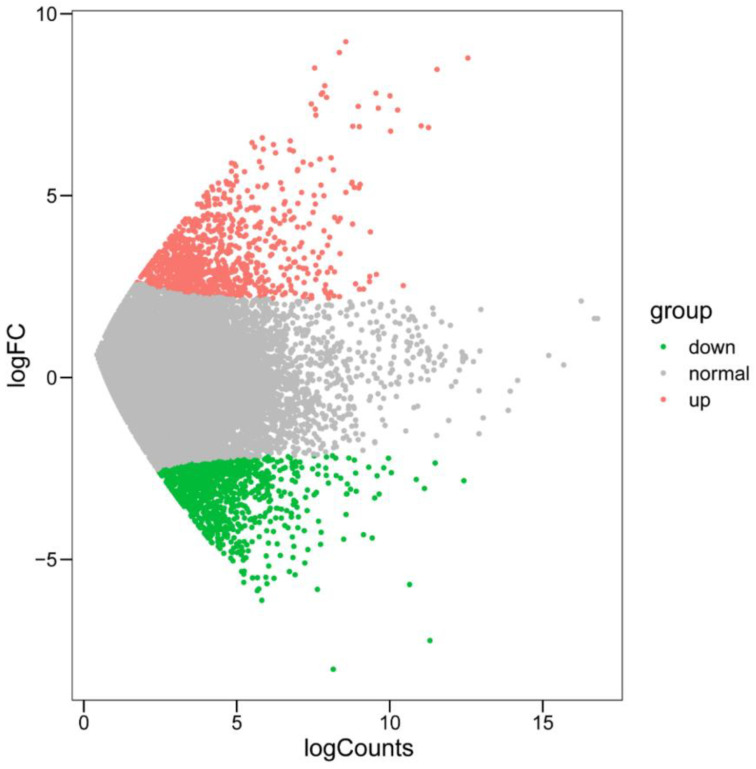
MA plot showing differentially expressed genes.

The analysis results showed that there were 1040 differentially expressed genes between the PCu and FCu (Figure 1). In total, 494 genes were downregulated and 546 genes were upregulated in the PCu compared with the FCu (Supplementary Table S1). Using a BLASTX search against the NR database, we found that the upregulated genes were mainly related to metabolism and parasitism (Table 2). Metabolic processes included the fatty acid metabolism, lipid metabolism, amino acid metabolism, TCA cycle, and mitochondrion carrier. Parasitism-related genes were mainly heat shock proteins (HSPs), protein kinases (PKs), leishmanolysin family protein, and actin (Table 3). Among them, HSPs play an important role in differentiation, cell cycle control, and immune and stress responses [34,35,36]. PKs help with the proliferation and differentiation of parasites [37]. Leishmanolysin and actin help invade into host cell when ciliatosis and other parasitic diseases occur [38,39]. Downregulated genes were related to repressors, open reading frame kinase (ROK), and aldo/keto reductase (AKRs). Other annotation results are shown in Supplementary Table S2.

**Table 2:** Significantly up- or downregulated expressed genes in the parasitic *C. uncinata* compared to the free-living *C. uncinata.*

	Up/Down	NR Description	Annotated Species
Fatty acid/lipid metabolism	up	putative fatty acid desaturase	*Trypanosoma theileri*
	up	oxysterol-binding protein	*Ichthyophthirius multifiliis*
Amino acid metabolism	up	glutamate/leucine/phenylalanine/valine dehydrogenase	*Tetrahymena thermophila*
	up	linear amide C-N hydrolase, choloylglycine hydrolase family protein	*Tetrahymena thermophila*
	up	serine carboxypeptidase family protein	*Tetrahymena thermophila*
	up	eukaryotic aspartyl protease	*Tetrahymena thermophila*
	up	Cyclophilinlike peptidyl-prolyl cis-trans isomerase domain	*Pseudocohnilembus persalinus*
TCA cycle and ETC	up	succinyl-CoA synthetase, alpha subunit, putative	*Ichthyophthirius multifiliis*
	up	2-oxoglutarate/malate translocase OMT	*Tetrahymena thermophila*
	up	isocitrate lyase	*Tetrahymena thermophila*
Mitochondrion carrier protein	up	ABC transporter family protein	*Tetrahymena thermophila*
	up	multidrug resistance proteinlike transporter family ABC domain protein	*Tetrahymena thermophila*
	up	transport protein	*Stylonychia lemnae*
Parasitism related	up	heat shock 70 kDa protein	*Tetrahymena thermophila*
	up	heat shock-binding protein 70, ER luminal protein	*Tetrahymena thermophila*
	up	heat shock protein HSP90	*Philasterides dicentrarchi*
	up	protein kinase	*lchthyophthirius multifiliis*
	up	leishmanolysin family protein	*Chilodonella uncinata*
	up	actin I	*Opisthonecta henneguyi*
	up	dynein heavy chain, amine-terminal region protein	*Tetrahymena thermophila*
ROK	down	ROK (repressor, open reading frame, kinase) family protein	*Tetrahymena thermophila*
AKRs	down	aldo/keto reductase family oxidoreductase	*Tetrahymena thermophila*

**Table 3:** Parasitism related genes characterized in protozoan parasites.

Gene	Function	Parasite	Disease	References
small heat shock proteins (sHSP) ORF-C4	response to stress	*Giardia lamblia*	Giardiasis	[40]
mitochondrial member of the HSP70 (mtp 70)	mitochondrial DNA replication	*Trypanosoma cruzi*	Chagas’ Disease	[41]
sHSP16	response to heat stress	*Trypanosoma cruzi*	Chagas’ Disease	[42]
HSP90	differentiation, cell cycle control	*Leishmania donovani*	Leishmaniasis	[36]
HSP100	promote amastigote development	*Leishmania donovani*	Leishmaniasis	[43]
HSP20	antigen for host immune system	*Leishmania amazonensis*	Leishmaniasis	[35]
HSP70	B cell mitogen	*Toxoplasma gondii*	Toxoplasmosis	[44]
HSP70	virulence factor	*Toxoplasma gondii*	Toxoplasmosis	[45]
HSP70C	immune stimulation	*Cryptocaryon irritans*	Cryptocaryonosis	[34]
protein kinases (PKs)	proliferation and differentiation	*Plasmodium falciparum*	Malaria	[37]
leishmanolysin (MaPro 14)	helps invade into host tissue	*Philasterides dicentrarchi*	Scuticociliatosis	[39]
actin	invasion into host cell	*Trypanosoma cruzi*	Chagas’ Disease	[38]
actin	migration motility	*Toxoplasma gondii*	Toxoplasmosis	[46]

### 3.3. GO Enrichment of DEGs

**Figure 2:**
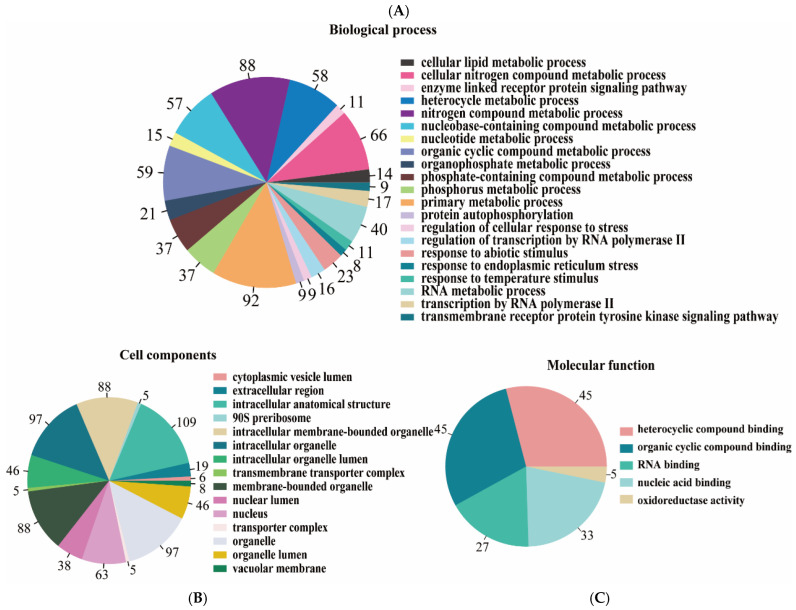
The GO enrichment of DEGs in the biological process category (**A**), cell components category (**B**), and molecular function category (**C**).

A total of 697 DEGs were assigned to the biological process category, 720 genes were assigned to the cell components category, and 155 genes were assigned to the molecular function category (Supplementary Table S3). Within the biological process category, the primary metabolic process and the nitrogen compound process were the most abundant subcategories (Figure 2A). Phosphorus, nitrogen, lipid, and protein were the main related metabolic substances. For the cellular component level, the intracellular anatomical structure and organelles were the main components (Figure 2B). Compared with these two levels, fewer genes were assigned to the molecular function level. Annotated genes were associated with the oxidoreductase activity, nucleic acid binding, and cyclic compound binding at this level (Figure 2C).

### 3.4. KEGG Pathway Annotation

**Figure 3:**
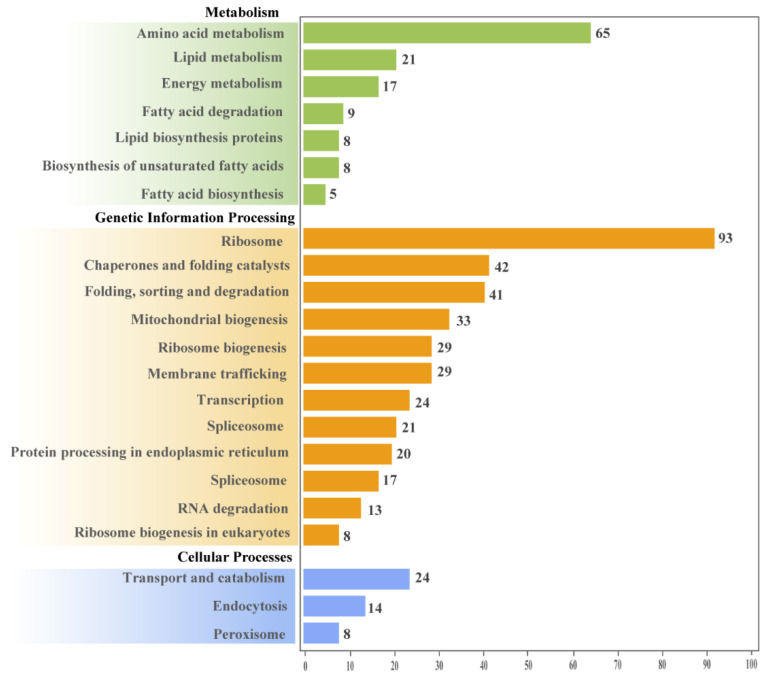
Number of DEGs annotated in different KEGG pathways.

In sum, 532 genes mapped onto KEGG pathways that were related to metabolism, genetic information processing, and cellular processes (Supplementary Table S4). Genetic information processing pathways had the most annotated genes compared to other pathways. In more detail, pathways related to ribosomes had the most annotated genes. As for metabolic pathways, amino acid metabolism, lipid metabolism, and energy metabolism were the top three metabolic pathways for which genes had been annotated. Pathways related to cellular processes had fewer annotated genes compared with the above two pathways (Figure 3).

### 3.5. Mitochondrial Proteins

**Figure 4:**
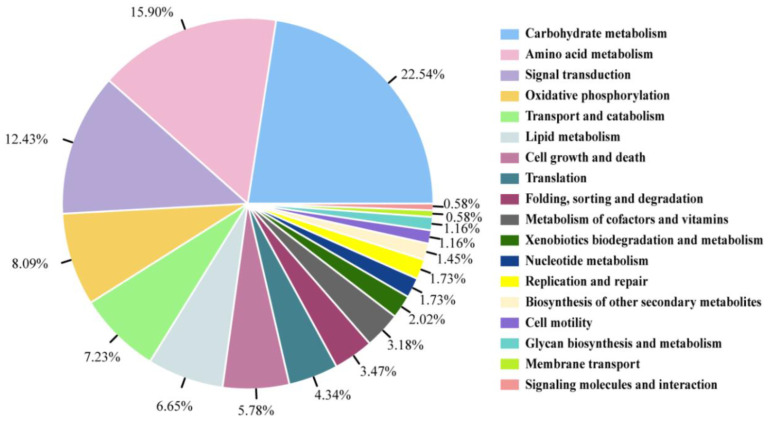
Functional distribution of the 346 mitochondrial proteins.

The BLASTP search results showed that 487 proteins had significant hits with mitochondrial proteomes of humans, *T. thermophila*, and other ciliates. Among these, 346 (71.04%) proteins were functionally annotated in KEGG. The main functions of these were carbohydrate metabolism, amino acid metabolism, signal transduction, oxidative phosphorylation, transport and catabolism, and lipid metabolism (Figure 4). These functions together accounted for >66% of the annotated entries. Among these, sequences containing N-terminal targeting peptides as predicted by TargetP and MitoFates were 89 and 72, respectively (Supplementary Table S5). CAI scores of the annotated genes are shown in Supplementary Table S6, and the normalized frequency distribution plot of CAI scores is shown in Supplementary Figure S1.

In more detail, electron transport chain (ETC) proteins, such as NADH dehydrogenase ubiquinone (NDU) subunit, succinate dehydrogenase, and cytochrome c oxidase (COX) subunit, were assigned. The representative protein also included the components of the mitochondrial transcription and translation machinery, such as the large subunit ribosomal protein (rpl) and small subunit ribosomal protein (rps), and the carrier proteins, such as the mitochondrial translocase of the outer membrane (TOM) subunits and mitochondrial translocase of the inner membrane (TIM) (Supplementary Table S5).

## 4. Discussion

Chilodonellosis is a disease of freshwater fish, caused by *Chilodonella* species, that causes serious economic losses in aquaculture [2,47]. Recently, evidence emerged that *C. uncinata* can be a fish pathogen in certain circumstances [11]. Results showed that *C. uncinata* does parasitize on gills and fins causing tissue damage and even host death. The goldfish infection model is easy to manage and apply in the laboratory. More experiments about drug screening and parasite prevention and control can be carried out based on this model in the future. Moreover, *C. uncinata* is a good model to study the mechanisms underlying the transition from a free-living to a parasitic lifestyle, considering its unique capacity of facultative parasitism. This study focuses on finding novel ways to manage chilodonellosis in aquaculture and screening the potential key genes related to parasitism. Gene expression and energy metabolism of parasitic and free-living *C. uncinata* were also explored herein.

Differentially expressed genes between free-living *C. uncinata* and parasitic *C. uncinata* were mainly assigned to metabolism and parasitism. The closest matches were the corresponding genes in *I. multifiliis* and *T. thermophila*. *I. multifiliis* is an obligate parasitic ciliate causing white spot disease in freshwater fish [48], while *T. thermophila* is a free-living ciliate that is a model organism for molecular and cellular biology [49,50,51]. The annotated DEGs indicated the existence of significant transcriptomic differences between the parasitic and free-living *C. uncinata*.

There are many reports about the parasitism-related genes in protozoan parasites (Table 3). In this study, compared with the free-living *C. uncinata*, the parasitic *C. uncinata* had a higher expression of actin and dynein heavy chain. Actin is a cytoskeletal protein which is ubiquitous in eukaryotes [52], and required for normal ciliary motility and phagocytosis [53]. In the model ciliates *Tetrahymena* and *Paramecium*, actin was detected at the oral apparatus [54,55], food vacuoles [56], cytoproct [57], basal bodies [54,55], and cilia [51,54,58]. In some parasitic protozoans, actin plays an important role in invasion and parasitism. For example, it was shown that actin was involved in the invasion into the host cells of *Trypanosoma cruzi* [38]. Additionally, apicomplexan parasites employ gliding motility that depends on the polymerization of parasite actin filaments for the host cell entry [46]. The higher expression of actin in the parasitic *C. uncinata* may also indicate that actin was involved in the host invasion.

Dynein is a well-characterized cytoskeletal motor, as its heavy chain component can produce movement. The heavy chain folds into a globular head that contains the motor activity and an extended tail that mediates the tethering of the motor to its cargo [59]. The heavy chain of dynein contributes to the initiation and propagation of ciliary bending [60]. Parasitic protozoa depend on active motility to colonize host tissues, escape host defenses, or reach locations within the host and enable transmission [61]. We speculate that parasitic *C. uncinata* needs to be adsorbed to the gill or body surface of the host to prevent it from being washed away by the water flow generated by the host movement and to colonize the gills and fins.

The parasitic *C. uncinata* had a higher expression of HSP70 and HSP90 compared with the free-living *C. uncinata*. It was reported that many protozoan parasites increase the expression of HSPs under exposure to environmental stress, such as elevated temperature, chemical stress, and host immune response [62,63]. These parasites include *Trypanosoma cruzi*, *Plasmodium falciparum*, *Leishmania donovani*, etc., that can cause serious diseases such as malaria, trypanosomiasis, and leishmaniasis [64]. Based on the previous reports, it can be inferred that the upregulated HSPs of parasitic *C. uncinata* may be a reflection of its resistance to the host immune response. At the same time, there is a report showing that the immobilization antigen vaccine adjuvanted by HSP70 from *Cryptocaryon irritans* could provide protection to grouper *Epinephelus coioides* against cryptocaryonosis [34]. From this perspective, HSP70 can also be taken into account when designing vaccines against *C. uncinata* in the future. Except for the proteins mentioned above, the expression of other parasitism-related proteins such as protein kinases (PKs) and leishmanolysin was also higher in the parasitic than in free-living *C. uncinata*. These proteins may play a role in the adaptation to the parasitic lifestyle in *C. uncinata*. More molecular experiments need to be done separately to explore their functions in the future.

Consistent with the NR annotation results, DEGs revealed significant enrichment in the amino acid metabolism in both GO terms and KEGG pathways. Specifically, glutamate dehydrogenase (GDH) was upregulated. GDH is found in all living organisms. It catalyzes the oxidative deamination of l-glutamate to α-KG using NAD(P)+ as a coenzyme [65]. GDH is also an anabolic enzyme catalyzing the assimilation of ammonia by the ruminal ciliate protozoan *Entodinium caudatum* [66]. Except for the amino acid metabolism, genes related to fatty acid/lipid metabolism were also strongly upregulated (Table 2). GO and KEGG annotation results also showed the differences in the phosphorous metabolic process and fatty acid/lipid biosynthesis. Lipids are essential and highly abundant components of all organisms. They serve as the main building blocks of the membranes that surround and compartmentalize cells, as a store of energy, and they regulate the localization and function of many proteins [67]. Lipids are also an important pathogenesis factor for parasites such as the malarial parasite apicomplexans [68]. Further studies are needed to determine whether lipids play a role in adaptation to parasitism in *C. uncinata*.

All metabolic pathways mentioned above are associated with energy transformation, and mitochondria play a crucial role in energy production. Two of the most critical mitochondrial functions are the tricarboxylic acid (TCA) cycle and oxidative phosphorylation. Herein, the genes of the parasitic *C. uncinata* for the TCA cycle were upregulated, which may indicate a faster metabolic rate than the free-living *C. uncinata*, while the expression of mitochondrial proteins did not significantly differ between the parasitic and free-living *C. uncinata*. This may indicate that the main metabolic pathways were not affected by the lifestyle (parasitic vs. free-living) in *C. uncinata*. Except for the metabolic processes, other pathways, such as cell components, molecular function and genetic processes were also enriched (Figure 2 and Figure 3). This suggests that the influences of different lifestyles are reflected in various aspects of the transcriptome of *C. uncinata*. Thus, to better facilitate the development of effective prevention and control strategies for Chilodonellosis, we should know more about the basic biological characteristics of *C. uncinata*, such as its life cycle, behavior, metabolism, physiology, and host recognition. Additionally, multiple research methods are critically needed in the near future to reveal the relevant mechanisms mentioned above, which is also applicable for other fish parasitic diseases.

## 5. Conclusions

In the light of recent reports of its facultative parasitism and impacts to aquaculture [10,11], *C. uncinata* presents a good model to research the impacts of parasitic lifestyle on the physiology and metabolism. Herein, the transcriptomes of parasitic and free-living *C. uncinata* were sequenced, analyzed, and compared. Multiple parasitism-related genes such as HSPs, actin, and leishmanolysin were upregulated in parasitic *C. uncinata* in comparison to the free-living *C. uncinata*, so knockdown or deletion of these highly expressed genes related to parasitism may contribute to the prevention and treatment of chilodonelliasis in future research. Moreover, the significant differences in metabolic processes including the amino acid metabolism, lipid metabolism, TCA cycle, etc., between these two lifestyles also gives us a hint that metabolism is largely influenced by lifestyle. This work contributes to our understanding of the transcriptional and metabolic adaptation of *C. uncinata* to the parasitic lifestyle, and it also has great significance for our understanding of the adaptive mechanisms that allow adaptation from free-living to a parasitic lifestyle. Further researches about parasitism adaption need to be done in the near future.

## Figures and Tables

**Table 1:** Summary of the de novo assembly of transcriptomic profiles of *C. uncinata*.

Sample	Number of Reads	Total Length (bp)	Number of Contigs	Max Length (bp)	Average Length (bp)
PCu	16,359,946	24,013,505	41,584	14,589	610.30
FCu	15,592,937	21,266,069	40,236	11,712	584.09

## Data Availability

The datasets used and/or analyzed in the current study are available from the corresponding author on reasonable request.

## Supplementary Materials

The following supporting information can be downloaded at: https://www.mdpi.com/article/10.3390/microorganisms10081646/s1. Table S1: DEGs of the *C. uncinata*; Table S2: NR annotation of the DEGs; Table S3: GO enrichment analysis of DEGs; Table S4: KEGG pathways enrichment of DEGs; Table S5: Annotation of mitochondrial proteins; Table S6: CAI scores; Figure S1: Frequency distribution plot of CAI scores.
